# Categorizing Extremely Positive Five-Star Online Reviews for Orthopedic Foot and Ankle Surgeons: A Retrospective Study

**DOI:** 10.7759/cureus.71932

**Published:** 2024-10-20

**Authors:** Albert Anastasio, Anthony N Baumann, Lulla V Kiwinda, Lindsey V Ruderman, Kyle Hitchman, Andrew E Hanselman, Samuel B Adams

**Affiliations:** 1 Orthopedic Surgery, Duke University School of Medicine, Durham, USA; 2 Orthopedics, Northeast Ohio Medical University College of Medicine, Rootstown, USA; 3 Orthopedic Surgery, Campbell University School of Osteopathic Medicine, Lillington, USA

**Keywords:** elective foot and ankle surgery, five-star reviews, online reviews, patient satisfaction, physician ratings, physician review websites, value-based care

## Abstract

Background

Multiple studies have emphasized the increased use of physician rating websites by patients when searching for surgeons to perform elective procedures. This study aimed to analyze the comments associated with online five-star patient reviews for orthopedic foot and ankle surgeons.

Methods

A retrospective analysis of five-star online reviews and corresponding comments using Vitals.com in 2024 was completed. Surgeons were included if they could be found on Vitals.com, were within a 10-mile radius of one of the top 10 largest cities in the United States, and if they had at least one review with one corresponding comment. Comments were further stratified into the following categories: good outcomes, well-controlled pain, correct diagnosis, clear plan, bedside manner/patient experience, staff compliment, wait time, nice facility, and offering a nonsurgical option.

Results

In a sampling of 2,425 orthopedic foot and ankle surgeons, 148 physicians (6.1%) had at least one review with one comment. Ultimately, 1,833 five-star reviews comprising 3,215 comments were included in the final analysis. Comments stratified by category revealed the most common comments being related to good outcomes (n = 940; 29.2%) and bedside manner/patient experience (n = 921; 28.6%). From the comments related to bedside manner/patient experience (p < 0.001) and presence of a clear plan (p < 0.001), a significantly higher proportion of comments in the nonoperative group was found relative to the operative group. Conversely, from the comments related to well-controlled pain (p < 0.001), a significantly higher proportion of comments in the operative group was found relative to the nonoperative group.

Conclusions

The most common reasons behind five-star patient comments for orthopedic foot and ankle surgeons were related to good outcomes and bedside manner/patient experience. Comments from surgical patients were most likely to include mention of well-controlled pain, whereas comments from patients who underwent nonoperative care were more likely to center on bedside manner/patient experience and presence of a clear plan.

## Introduction

Increased emphasis on value-based care and patient satisfaction has been noted in recent years across orthopedic surgery [1]. With the passing of the Affordable Care Act, provisions were made to allow the public to access data on patient satisfaction with both hospital systems and specific providers [2]. In an effort to improve transparency within the provision of healthcare, government-funded websites such as HealthData.gov and the US Department of Health and Human Services Protect Public Data Hub have been created [3]. While well-intentioned, these websites remain relatively low-trafficked, and patients more commonly rely on public physician review websites (PRWs), such as Vitals, Yelp, Healthgrades, and Google reviews, when making decisions related to provider choice [4]. Indeed, 28.1% of patients have indicated that they “strongly agreed” that a positive physician review alone on a patient review website would cause them to seek care from that practitioner [5], Conversely, 27% of patients indicated that a negative review may result in hesitation to seek an appointment with that provider [5]. With the ability to access free-text comments and averaged provider scores (many sites utilize a ranking system from one- to five stars, which allows for easy comparison between providers) at the click of a button, PRWs likely will continue to be a major contributor to patient decision-making regarding surgical provider choice.

Given the demonstrated reliance on online reviews in driving patient selection, it becomes paramount to understand the underlying factors that are associated with strongly positive online reviews of orthopedic foot and ankle surgeons. While previous studies have analyzed strongly negative one-star reviews among orthopedic foot and ankle surgeons [6], none, to our knowledge, have yet investigated the components that contribute to an extremely positive (five-star) review. As the field expands and more elective indications for foot and ankle procedures are recognized, surgeon preference will continue to be of greater importance. Thus, the purpose of this study was to analyze the factors that contribute to a five-star review on PRWs among orthopedic foot and ankle surgeons.

## Materials and methods

Study creation

This was a retrospective analysis of five-star online reviews and corresponding comments by patients of foot and ankle orthopedic surgeons on Vitals.com, a popular physician rating website. Data collection took place between January and February 2024 and included all reviews that met inclusion criteria from the inception of Vitals.com to the date the physician’s profile was accessed. Reviews and comments for foot and ankle orthopedic surgeons (both MD and DO) were recorded from the top 10 largest cities in the US by population as found online (www.census.gov/newsroom/press-releases/2023/subcounty-metro-micro-estimates.html). All physicians from those cities within a 10-mile radius were examined on Vitals.com.

Inclusion criteria

Physicians were included if they could be found on Vitals.com, were within a 10-mile radius of one of the top 10 largest cities in the US, and if they had at least one review with one comment. Physicians were excluded if they did not have at least one review with one comment (i.e., if the physician had zero reviews or reviews without any comments), if they could not be found on Vitals.com, were not DO or MD, and were outside of a 10-mile radius from one of the top 10 largest cities in the US by population. Each individual review, from one- to five stars, was examined for each physician for possible inclusion.

Basic study definitions

For the purposes of this study, a “review” refers to the report, from one to five stars, found on Vitals.com. Subsequently, a “comment” refers to the custom text written by the patient on the review. For patient grouping, patients were grouped into the operative group or the nonoperative group. Patients were placed into either one or both groups based on whether they were patients who received surgical care or conservative care, based on the comment on the review. Patients who gave comments in which it was unclear if they received surgical care or conservative care were placed into the nonoperative group. Importantly, each review could have multiple comments based on the text found in the comments.

Comment category definitions

Comments were further stratified into the following categories based on content: good outcomes, well-controlled pain, correct diagnosis, clear plan, bedside manner/patient experience, staff compliment, wait time, nice facility, and offering a nonsurgical option. Well-controlled pain included comments related to pain that was lowered, controlled, managed, or improved by the physician. Good outcomes included any comment related to any kind of surgical or nonsurgical outcome, outside of well-controlled pain, reported by the patient. Correct diagnosis includes comments related to the patient’s belief that they were given the correct or proper diagnosis. A clear plan includes comments in which the patient reported that the physician gave a clear, concise, and/or understandable treatment plan. Bedside manner/patient experience included any kind of comment on the physician’s behavior (i.e., “really listened to me” or “was very kind and explained my condition to me”) or any general description of patient experience (i.e., “fantastic experience”). Staff compliment included comments related to hospital or clinic staff, apart from the treating physician, that were mentioned. Wait time included comments related to the wait time of the clinic. Nice facility included comments related to the building, supplies, or facility grounds. Offering a nonsurgical option refers to comments related to the physician offering conservative care options.

Data extraction

Data extraction was completed by two authors. Data included total physicians reviewed, total physicians with a least one review (of any star level) with at least one comment, total reviews (of any star level), total five-star reviews, total comments, and total comments by operative group status and comment category. 

Statistical considerations

IBM SPSS Statistics for Windows, Version 29.0 (Released 2022; IBM Corp., Armonk, NY, USA) was used for statistical analysis in this study. Descriptive statistics such as mean, median, standard deviation, and minimum-maximum with frequency reporting were used to describe the data. A chi-square test with two-sided p-values was used for comparison between two categorical variables, with p < 0.05 considered significant.

## Results

Study demographics

A total of 2,425 physicians were examined from the top 10 most populated cities in the United States. Of those 2,425 physicians, there were 148 physicians (6.1%) with at least one review (of any number of stars) with one comment. From those 148 physicians, there were 2,377 reviews with 1,833 five-star reviews (77.1%). The mean number of five-star reviews per physician (n = 148) was 12.4 ± 20.1 reviews (median: 6.0 reviews; minimum-maximum: 0.0-174.0 reviews). Out of those 1,833 five-star reviews, there were 3,215 comments, with 1,210 comments (37.6%) in the operative group and 2,005 comments (62.4%) in the nonoperative group. The mean number of comments per physician (n = 148) was 21.7 ± 36.0 comments (median: 11.0 comments; minimum-maximum: 0.0-323.0 comments) (Table 1).

**Table 1:** Study demographics for the present study

Demographic information	Values
Total physicians examined (n)	2,425 physicians
Physicians with at least one review with one comment (n)	148 physicians
Total one- to five-star reviews examined (n)	2,377 reviews
Total five-star reviews examined (n)	1,833 reviews
Mean five-star reviews per physician	12.4 ± 20.1 reviews
Median five-star reviews per physician	6.0 reviews
Total comments examined (n)	3,215 comments
Operative group comments (n)	1,210 comments
Nonoperative group comments (n)	2,005 comments
Mean comments per physician	21.7 ± 36.0 comments
Median comments per physician	11.0 comments

Comments on the five-star reviews

**Figure 1:**
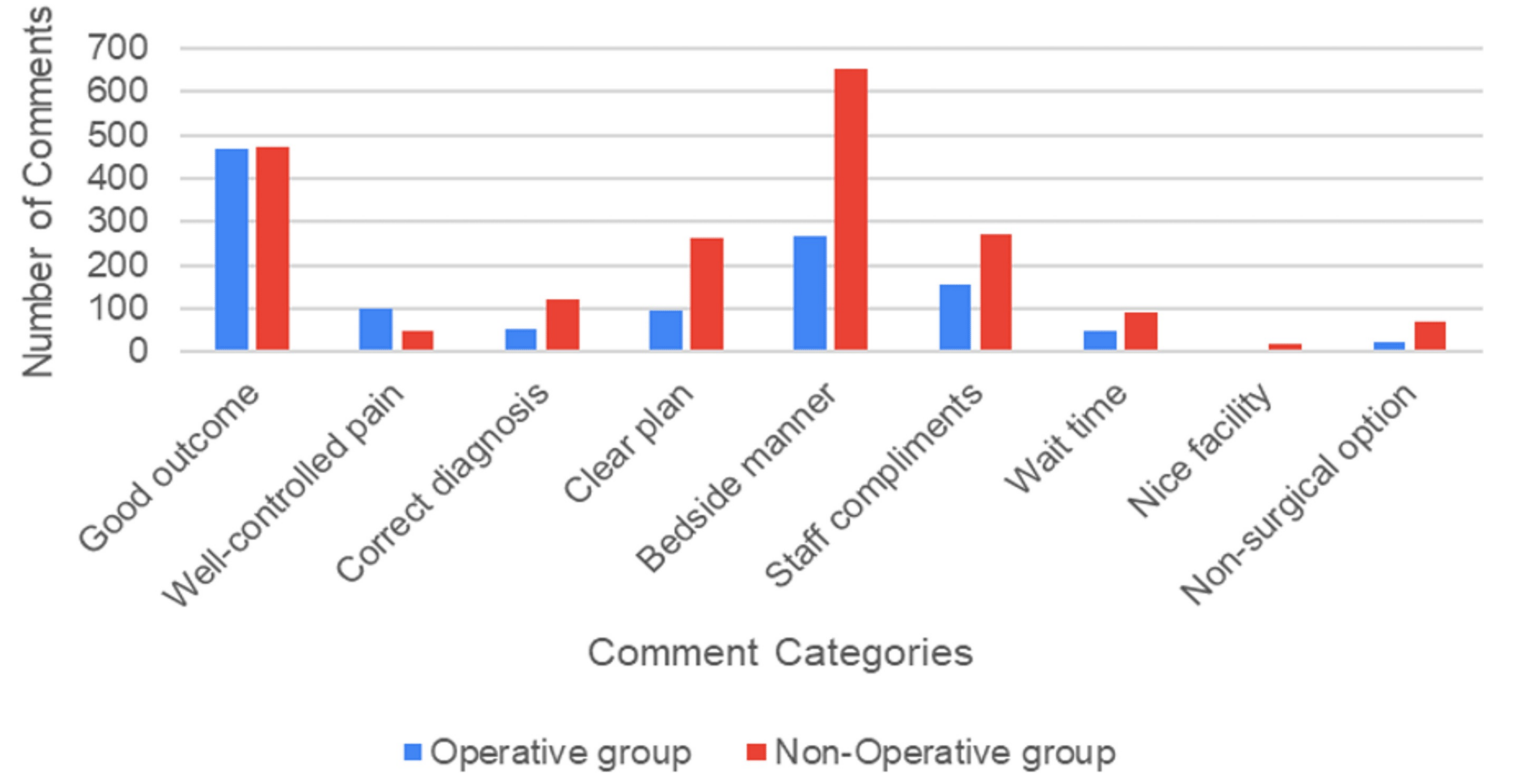
Comments from the five-star reviews stratified by categories (good outcome, well-controlled pain, correct diagnosis, clear plan, bedside manner, staff compliments, wait time, nice facility, and offering a nonsurgical option) and operative status (operative versus nonoperative group)

All five-star reviews (n = 3,215; 77.1% of total reviews) were stratified into categories: good outcomes, well-controlled pain, correct diagnosis, clear plan, bedside manner/patient experience, staff compliment, wait time, nice facility, and offering a nonsurgical option. Comments stratified by category revealed 29.2% related to good outcomes (n = 940), 4.5% related to well-controlled pain (n = 146), 5.4% related to a correct diagnosis (n = 174), 11.1% related to a clear plan (n = 357), 28.6% related to bedside manner/patient experience (n = 921), 13.3% related to staff compliments (n = 426), 4.2% related to wait time (n = 136), 0.7% related to a nice facility (n = 24), and 2.8% related to offering a nonsurgical option (n = 91) (Figure 1).

Comments by operative versus nonoperative category

From the 940 comments related to good outcomes, 467 comments (38.6% of operative group comments) were in the operative group and 473 comments (23.6% of nonoperative group comments) in the nonoperative group, with a significantly higher proportion of comments in the operative group (p < 0.001) (Table 2). From the 146 comments related to well-controlled pain, 100 comments (8.3% of operative group comments) were in the operative group, and 46 comments (2.3% of nonoperative comments) were in the nonoperative group, with a significantly higher proportion of comments in the operative group (p < 0.001). From the 174 comments related to a correct diagnosis, 53 comments (4.4% of operative group comments) were in the operative group and 121 comments (6.0% of nonoperative comments) were in the nonoperative group, with a significantly higher proportion of comments in the nonoperative group (p = 0.045). From the 357 comments related to a clear plan, 94 comments (7.8% of operative group comments) were in the operative group, and 263 comments (13.1% of nonoperative group comments) were in the nonoperative group, with a significantly higher proportion of comments in the nonoperative group (p < 0.001). From the 921 comments related to bedside manner/patient experience, 267 comments (22.1% of operative group comments) were in the operative group, and 654 comments (32.6% of nonoperative group comments) were in the nonoperative group, with a significantly higher proportion of comments in the nonoperative group (p < 0.001). From the 426 comments related to staff compliments, 155 comments (12.8% of operative group comments) were in the operative group and 271 comments (13.5% of nonoperative comments) were in the nonoperative group, with no significant difference in proportions between groups (p = 0.567). From the 136 comments related to wait time, 47 comments (3.9% of operative group comments) were in the operative group and 89 comments (4.4% of nonoperative group comments) were in the nonoperative group, with no significant difference in proportions between groups (p = 0.449). From the 24 comments related to a nice facility, five comments (0.4% of operative group comments) were in the operative group and 19 comments (0.9% of nonoperative group comments) were in the nonoperative group, with no significant difference in proportions between groups (p = 0.088). From the 91 comments related to offering a nonsurgical option, 22 comments (1.8% of operative group comments) were in the operative group and 69 comments (3.4% of nonoperative group comments) were in the nonoperative comments, with a significantly higher proportion of comments in the nonoperative group (p = 0.007).

**Table 2:** Comment stratification by operative group versus nonoperative group with p-values

Comment categories	Operative group	Nonoperative group	p-values
Good outcome	467 (38.6%)	473 (23.6%)	p < 0.001
Well-controlled pain	100 (8.3%)	46 (2.3%)	p < 0.001
Correct diagnosis	53 (4.4%)	121 (6.0%)	p = 0.045
Clear plan	94 (7.8%)	263 (13.1%)	p < 0.001
Bedside manner	267 (22.1%)	654 (32.6%)	p < 0.001
Staff compliments	155 (12.8%)	271 (13.5%)	p = 0.567
Wait time	47 (3.9%)	89 (4.4%)	p = 0.449
Nice facility	5 (0.4%)	19 (0.9%)	p = 0.088
Nonsurgical option	22 (1.8%)	69 (3.4%)	p = 0.007
Total comments	1,210 (100.0%)	2,005 (100.0%)	-

## Discussion

The goal of this study was to evaluate the elements that lead a patient to leave a strongly positive review of an orthopedic foot and ankle surgeon on a PRW. The most common reasons behind five-star patient comments for orthopedic foot and ankle surgeons were related to good outcomes and bedside manner/patient experience in both operative and nonoperative groups of patients. Comments from patients who underwent operative care were most likely to include mention of well-controlled pain and good outcomes, whereas comments from patients who underwent nonoperative care were more likely to center on bedside manner/patient experience and presence of a clear plan.

Orthopedic foot and ankle surgeons treat high volumes of patients for both operative and nonoperative conditions. Surgeons should, therefore, be aware of the different factors that contribute to positive patient experiences in each of these settings and take this into consideration in their practice. For example, surgeons may consider specific focus during a nonsurgical patient visit on outlining a clear plan of conservative intervention with appropriate follow-up. Providers may consider utilizing effective communication strategies such as the “teach back” method to ensure patients understand the nonoperative intervention strategy [7-11]. Likewise, specific approaches for surgically treated patients may result in higher patient satisfaction. For example, given patient focus on well-controlled pain in five-star reviews for operative foot and ankle patients, emphasis on compassionate care for patients dealing with pain issues post-surgically may actually improve patient perception of the degree of pain [12-14]. In summation, a better understanding of what specific patients are searching for in their orthopedic foot and ankle care should empower surgeons to feel more in control of their ability to positively impact their average review on PRWs and, more importantly, to more optimally treat their patients.

While no study to date has evaluated five-star reviews in orthopedic foot and ankle surgery, previous studies have examined positive reviews in other fields [15]. Noel et al. pulled five-star reviews of orthopedic sports surgeons from PRWs and examined the free-text portions of the reviews for factors related to excellent reviews within orthopedic sports medicine [16]. These authors report a high emphasis on five-star reviews on nonclinical compliments such as good bedside manner, professional/friendly staff, and ease of scheduling. In direct agreement with the present study, patients who were treated with surgery tended to focus on good outcomes and control of pain [16]. Other authors have explored the rationale behind negative reviews on PRWs [17]. Overwhelmingly, interpersonal skills and bedside manner are cited as major factors behind extremely negative, one-star reviews [18-23]. Other considerations such as financial concerns and unprofessional staff [19], as well as prolonged wait time [24] are reported regularly as contributing factors to patient dissatisfaction. While perhaps intuitive, these collective findings further confirm the notion that excellent bedside manners and a professional and friendly staff team are paramount to patient satisfaction in orthopedic surgery.

PRWs appear to be a mainstay for patient surgeon choice, despite many concerns regarding the legitimacy of such websites and the reviews contained within. For example, up to 20% of the reviews on other PRWs have been identified as being fraudulent [20,25] Several authors have identified reviews on PRWs that were written by physicians themselves [26]. A strong incentive for hospital corporations and practice groups to post fabricated positive reviews exists. Conversely, in an increasingly competitive healthcare climate and in an era of declining reimbursements, certain groups may be incentivized to produce fraudulent negative reviews for popular competing surgeons within their catchment area. To combat the duplicitous use of PRWs, modern artificial intelligence (AI) models have shown promise in identifying fake online reviews. Convolutional neural networks in combination with adaptive particle swarm optimization with natural language processing techniques have been shown to outperform traditional machine learning modalities in identifying fake online reviews, with an impressive accuracy rate of 99.4% [25]. Other AI modalities have also been applied to this end [27-29]. In summary, further improvement of the quality and accuracy of information available on PRWs will empower patients to make more informed decisions while also providing assurance to providers regarding their efforts towards empathetic care.

Several limitations should be considered in the interpretation of this manuscript. A strong potential for selection bias is present in this study, given the use of only one PRW and our choice to utilize the top 10 most populous cities in the US for surgeon selection. Other PRWs may reveal differing trends in patient comments regarding positive reviews. Similarly, there is also potential for misclassification bias as categorization was based on the author’s interpretation of the patient’s written comments. However, we attempted to create numerous categories to fully encapsulate the diversity of patient comments on five-star reviews. Another limitation of the present study is the subjective nature inherent to patient reviews of physicians. For example, the present study was unable to confirm diagnoses for comments that were classified as mentioning a correct diagnosis. However, the authors were consistent in the categorization of comments that mentioned having received previous diagnoses and treatments that did not alleviate their issue, and the physician that they were commenting on gave them what they believed to be a correct diagnosis and associated treatment that did alleviate their issue. The subjective nature of these comments is not unique to the present study but is a limitation of other similar studies that have analyzed online reviews from PRWs [6,17,21-24]. Another limitation is that patients living outside of major US metropolitan areas may emphasize other aspects of care than those reported in this manuscript as major contributors to patient satisfaction. Further research should evaluate other PRWs and seek to explore geographical variation in patient satisfaction, such that surgeons practicing in specific regions can more appropriately understand how to optimally serve their patients. Finally, this study was limited by a small sample size in some of the comment categories, although this may be reflective of comment rarity as a lot of comments were reviewed by a large number of foot and ankle orthopedic surgeons.

## Conclusions

The most common comments related to five-star patient reviews for orthopedic foot and ankle surgeons mentioned good outcomes and bedside manner/patient experience. Comments from patients who underwent operative care were most likely to include reference to well-controlled pain, whereas comments from patients who underwent nonoperative care were more likely to center on bedside manner/patient experience and presence of a clear plan.
